# Examining the Association Between Differences in Post Turning Driving Behavior on a Driving Simulator and Amyloid Status in Cognitively Normal Older Adults

**DOI:** 10.1002/alz.089501

**Published:** 2025-01-03

**Authors:** Savannah G. Rose, Emma Flynn, Douglas Carmichael, Yi Lu Murphy, Robert A. Koeppe, Carol C. Persad, Amanda Cook Maher, Bruno Giordani

**Affiliations:** ^1^ University of Michigan, Ann Arbor, MI USA; ^2^ University of Michigan ‐ Dearborn, Dearborn, MI USA; ^3^ University of Michigan Medical School, Ann Arbor, MI USA

## Abstract

**Background:**

Older adults diagnosed with Mild Cognitive Impairment (MCI) and dementia are at increased risk of driving impairment. As the prevalence of dementia, particularly Alzheimer’s dementia, increases, researching the link between driving behaviors and cognitive decline may aid early identification of at‐risk cognitively normal older adults. This study assesses differences in driving behavior following a left hand turn between amyloid positive and negative cognitively normal older adults.

**Method:**

Seventy‐three cognitive normal adults aged 65+ were categorized as either amyloid positive or amyloid negative based on PiB PET imaging and a centiloid scale (**≥**20 = positive, **≤**10 = negative). Forty‐three participants were amyloid negative (CN‐) and 27 were positive (CN+). All participants completed a 20‐minute simulator drive programmed with realistic driving scenarios, including a 90 degree left turn from a stop sign at two intersecting rural two lane roads. Assessed turning behavior in the 120 meters after the left turn included the average absolute value of lane deviation, number of off‐road errors and oncoming traffic errors, number of over‐corrections, and number of total errors. Group differences were analyzed using independent samples t‐tests.

**Result:**

There were no demographic differences between the groups. The CN‐ group had faster speed after the turn, faster average speed in the 120m post‐turn, and greater average absolute value lane offset compared to the CN+ group. The CN‐ group also made more errors, including both off‐road and oncoming‐traffic error types, compared to the CN+ group. There was no significant difference between the number of over corrections or total errors by group.

**Conclusion:**

Overall, cognitively normal older adults with elevated brain amyloid displayed potentially more cautious driving behavior than their peers without elevated amyloid. It remains to be seen whether these more cautious behaviors are potentially due to decreased confidence or subconscious recognition of cognitive changes. Nonetheless, they may signal early, subtle signs of AD‐related processes differentiating individuals at‐risk for AD‐related cognitive decline.

